# Effects of Bacillus pumilus precipitation on the flexural strength of jute fibre reinforced concrete

**DOI:** 10.1038/s41598-025-34050-y

**Published:** 2025-12-30

**Authors:** Uwemedimo Nyong Wilson, Abraham Salami, Mohammed Abdulkareem Adisa

**Affiliations:** 1 Department of Civil Engineering, Nigerian Defence Academy, Kaduna, Nigeria; 2 Department of Civil and Environmental Engineering, Kwara State University, Malete, Kwara State Nigeria

**Keywords:** Bacillus pumilus, Microbially induced calcite precipitation (MICP), Jute fibre, Flexural strength, Self-healing concrete, Durability, Bacterial dosage, Tukey HSD, Engineering, Materials science, Microbiology

## Abstract

This study investigates the influence of *Bacillus pumilus*–induced calcium carbonate precipitation (MICP) on the flexural performance and durability of jute fibre–reinforced concrete (JFRC). A nominal 1:2:4 concrete mix with 1% jute fibre (treated and untreated) was prepared and dosed with three bacterial concentrations (B1.5, B12, and B24). Prismatic beams (150 × 150 × 500 mm) were cured and tested at 7, 14, 21, and 28 days under three-point bending, and a total of twelve beams per mix (*n* = 12), corresponding to three replicate specimens at each curing age, were evaluated. Fresh properties (slump and compaction factor), mass and dimensional loss, SEM microstructural observations, and statistical analysis (two-way ANOVA and Tukey HSD) were used to interpret results. Findings show that bacterial dosage strongly governs performance: low dosage (B1.5) produced minor early-age gains; moderate dosage (B12) yielded delayed but measurable improvements; and high dosage (B24) produced the greatest 28-day flexural enhancement, although with reduced workability. Durability tests indicated improved resistance to acid attack and lower mass and dimensional loss for bacterial mixes, with B1.5 and B24 dosages showing the most favourable performance depending on the metric assessed. SEM observations confirmed progressive CaCO₃ deposition with increasing bacterial concentration, enhancing fibre–matrix bonding and reducing microcrack connectivity. Overall, the study demonstrates a measurable synergy between jute fibres and microbially induced calcite precipitation, indicating that appropriately dosed *B. pumilus* can significantly enhance the flexural behaviour and durability of JFRC. These findings provide insight into the development of low-cost, bio-enhanced natural-fibre composites for sustainable construction applications.

## Introduction

**Fig. 1:**
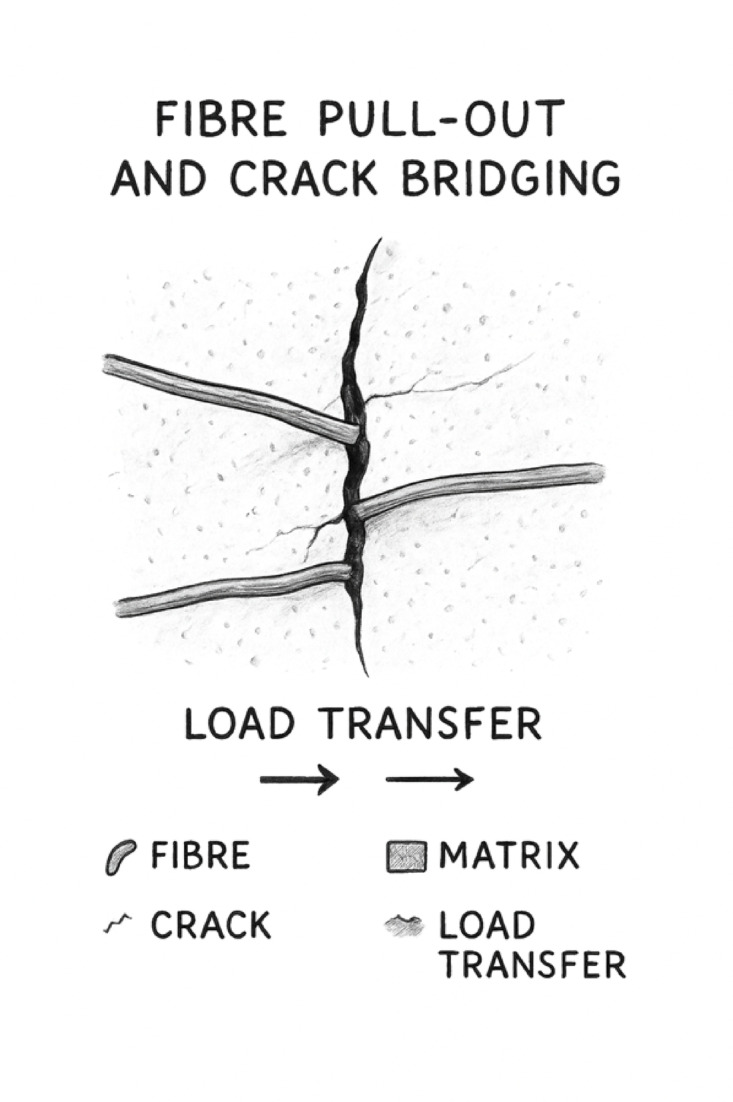
Illustration of fibre pull-out and crack bridging.

Concrete remains the most extensively used construction material worldwide owing to its high compressive strength, adaptability, and the ready availability of its raw materials^1^. However, despite its merits, conventional concrete is inherently brittle and exhibits low tensile and flexural strength, often leading to sudden failure under service conditions. To address these weaknesses, fibres are commonly incorporated into the cementitious matrix to form fibre-reinforced concrete (FRC), which enhances ductility, restricts crack propagation, and improves energy absorption by bridging microcracks and redistributing stresses^2^. In recent years, there has been growing interest in the use of natural fibres as eco-friendly and cost-effective alternatives to synthetic fibres in FRC. Natural fibres such as jute, coir, and sisal offer advantages of renewability, biodegradability, and local availability, thereby contributing to sustainable construction practices^3^. Among various natural fibres used in cementitious composites, jute fibre stands out due to its high tensile strength (400–800 MPa), moderate young’s modulus (10–30 GPa), low density (1.3–1.5 g/cm³), and high cellulose content (60–65%), which contribute to improved crack resistance and energy absorption in cement matrices^4–7^. Additionally, jute is abundantly available in tropical and subtropical regions, particularly in countries such as Bangladesh, India, and parts of Nigeria, where its cultivation is both economical and sustainable^8–10^. Beyond sustainability and availability, jute’s chemical composition (high cellulose and surface hydroxyl groups) and natural fibrillar microstructure are expected to provide abundant nucleation sites and improve mechanical interlocking with mineral precipitates, potentially promoting interfacial filling in bio-modified matrices. Nevertheless, the use of natural fibres presents challenges such as poor fibre–matrix adhesion, high moisture absorption, and degradation in alkaline environments, all of which reduce the mechanical efficiency and long-term performance of natural fibre reinforced concretes^11,12^. Consequently, while natural fibres improve the initial toughness and crack resistance of concrete, their effectiveness often diminishes over time due to interfacial deterioration, as illustrated in Fig. 1, where fibre pull-out and reduced crack-bridging capacity become evident.

**Fig. 2:**
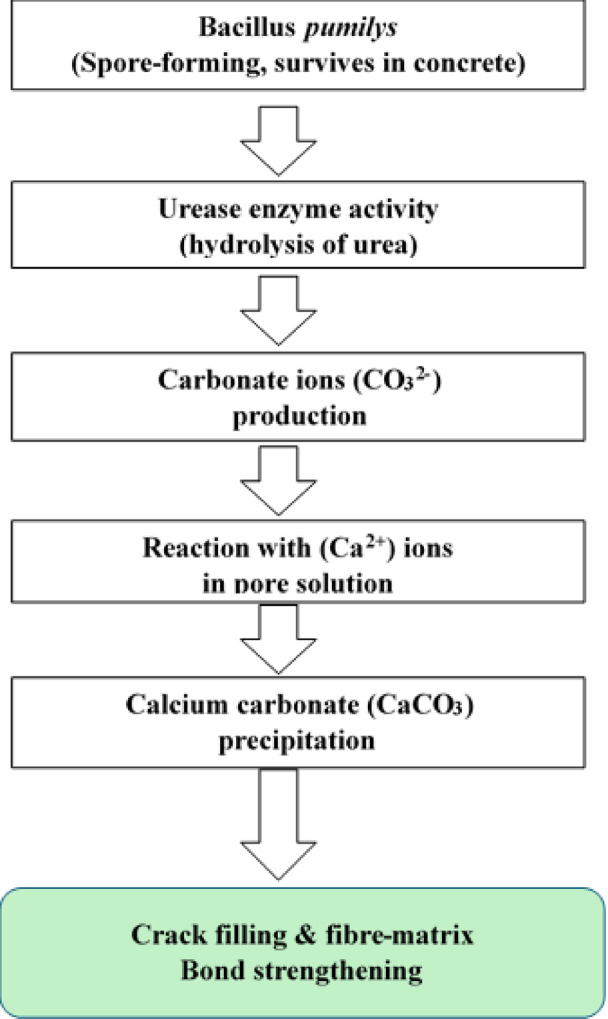
Mechanism of bacillus pumilus in bioconcrete.

Several surface modification and chemical treatment methods have been explored to overcome these limitations; however, many are costly, energy-intensive, or environmentally unsustainable. In contrast, microbially induced calcite precipitation (MICP) has emerged as a promising bio-based technique for improving the performance of cementitious materials^13–18^. MICP relies on ureolytic bacteria that hydrolyze urea to generate carbonate ions, which react with calcium ions in the matrix to form calcium carbonate (CaCO₃) precipitates. These precipitates fill pores, seal microcracks, and reinforce the interfacial transition zone (ITZ), thereby enhancing both strength and durability^19,20^. The approach is environmentally friendly and supports the advancement of self-healing and bio-cementation technologies within the broader context of sustainable materials development. Among the various ureolytic bacteria studied, Bacillus pumilus has shown remarkable promise due to its spore-forming ability, enzyme activity, and tolerance to highly alkaline environments typical of concrete. Its resilience and capacity to promote efficient CaCO₃ deposition make it particularly suitable for cement-based applications^21,22^. The overall process by which B. pumilus contributes to strength and durability in bioconcrete is illustrated in Fig. 2. The bacterium survives in the concrete matrix through spore formation, produces urease to hydrolyze urea, generates carbonate ions, and reacts with available calcium ions to precipitate CaCO₃ crystals. These crystals fill pores and microcracks, thereby enhancing durability and improving fibre–matrix bonding.

While several studies have investigated microbial self-healing and crack repair mechanisms, numerous researchers have demonstrated that bacterial incorporation with various synthetic and natural fibres can significantly enhance crack closure, flexural recovery, and overall durability of cementitious composites. For instance, Snoeck and De Belie^23^ reported up to 70% crack closure using microfibres with SAPs, while Wiktor and Jonkers^24^ achieved 90% crack sealing and 25% flexural stiffness recovery with polypropylene fibres and Bacillus subtilis. Similar improvements have been observed in PVA, glass, basalt, and hybrid fibre systems inoculated with bacterial strains such as B. sphaericus and B. pumilus, resulting in 20–40% increases in flexural strength and up to 90% microcrack healing^25,26^. Unlike sisal, PVA, or glass fibres studied previously, jute’s high cellulose content and abundant surface hydroxyl groups may provide additional nucleation sites for CaCO₃, potentially strengthening the fibre–matrix interface, a mechanism that has not yet been experimentally verified. Furthermore, although B. subtilis and S. pasteurii are commonly used MICP strains, B. pumilus offers higher alkaline tolerance and spore-forming resilience that may improve viability in fibre-reinforced matrices, yet its interaction with natural fibres remains unexplored. Despite these advancements, there remains limited understanding of how Bacillus pumilus-induced calcite precipitation can be harnessed to enhance the flexural behaviour and durability of jute fibre reinforced concrete (JFRC). This gap presents a valuable opportunity to integrate biological modification with natural fibre reinforcement to develop sustainable, high-performance concrete composites.

The present study therefore investigates the effect of Bacillus pumilus-induced calcite precipitation on the flexural strength and durability performance of jute fibre reinforced concrete. Specifically, it aims to characterize the properties of jute fibre and bacterial precipitates, evaluate the impact of calcite deposition on flexural strength, determine the optimal microbial dosage for maximum performance, and assess durability under acid attack through mechanical and microstructural analyses (SEM). The study hypothesizes that Bacillus pumilus-induced calcite precipitation improves the 28-day flexural strength of JFRC compared with untreated control specimens. The significance of this research lies in its contribution to the development of bio-enhanced, low-carbon cementitious materials that combine renewable natural fibres with microbial modification for improved mechanical and durability performance. The findings will provide insights into the potential of bio-calcification to strengthen fibre–matrix interfaces, reduce degradation, and extend service life. From an environmental standpoint, this approach supports global efforts to decarbonize the construction sector by reducing reliance on synthetic fibres and chemical additives. Moreover, the outcomes have practical implications for developing regions where jute is locally available and cost-effective construction materials are urgently needed. By integrating natural fibre reinforcement with MICP, the study advances a sustainable pathway toward durable, eco-friendly, and resource-efficient construction materials that align with circular economy principles and modern green building standards.

## Methodology

### Materials

**Fig. 3:**
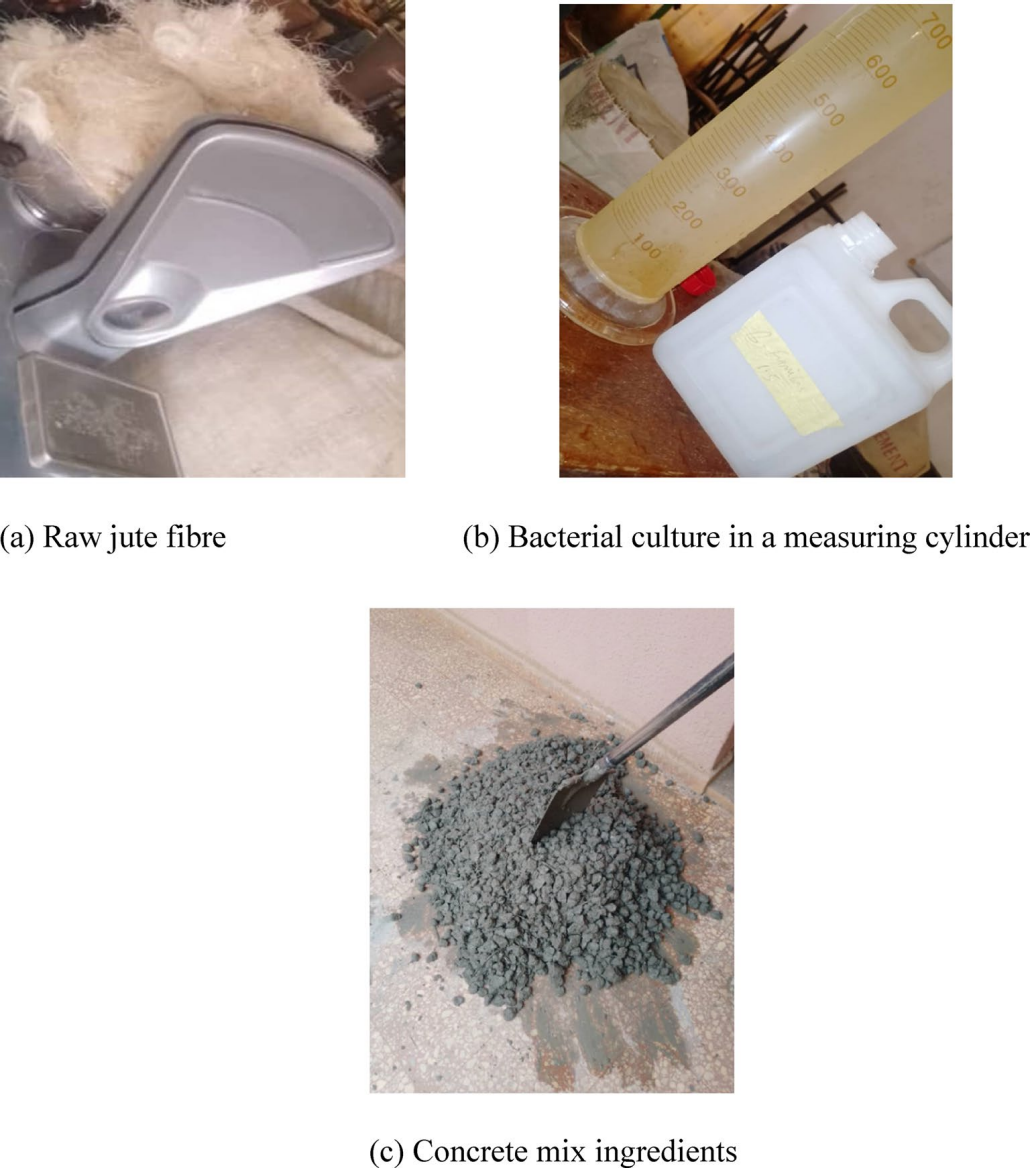
Materials.

The materials used in this investigation comprised ordinary Portland cement, fine and coarse aggregates, natural jute fibres, bacterial cultures, and clean water (Fig. 3). A Grade 42.5R ordinary Portland cement conforming to ASTM C150 and BS EN 197-1 standards was used throughout the study. The fine aggregate was river sand with a maximum particle size of 4.75 mm, tested for grading and specific gravity in accordance with ASTM C136 to ensure compliance with standard requirements. Crushed granite of 20 mm nominal size served as the coarse aggregate; it was well-graded to promote compaction, reduce voids, and achieve uniform strength development in the concrete. Natural jute fibres were locally obtained and cut into lengths of 20–30 mm before incorporation into the mix, its properties shown in Table 1. Both untreated and treated forms of the fibres were used to assess the influence of surface condition on performance. Jute fibres were selected because of their high tensile strength, moderate stiffness, and abundant availability in tropical regions, making them an economical and sustainable reinforcement material. The bacterial component consisted of Bacillus pumilus, selected for its ability to induce calcium carbonate precipitation and promote self-healing in cementitious matrices. The bacterial culture was grown in nutrient broth and prepared at three defined cell densities—1.5 × 10⁸, 12 × 10⁸, and 24 × 10⁸ cells/mL—coded as B1.5, B12, and B24, respectively, to avoid misinterpretation as percentage values^27,28^. Potable water free from impurities was used for mixing and curing in accordance with ASTM C1602. All materials were carefully handled and stored to prevent contamination or variation in quality throughout the experimental program.

**Table 1:** Properties of jute fibre.

Sample	Tensile Strength (MPa)	Young Modulus (MPa)	Elongation (%)
A	365	0.0107	12.9
B	355	0.01	12
C	362	0.01	11
**Average**	**360.67**	**0.0102**	**—**

### Mix proportions

A nominal 1:2:4 concrete mix (cement: fine aggregate: coarse aggregate) with a water–cement ratio of 0.55 was used following standard guidelines^29^. The control mix contained no fibres or bacterial culture. Jute fibre–reinforced mixes incorporated 1% fibre by weight of cement in untreated and treated forms^30^. The control mix contained no jute fibres and no bacteria, serving as the baseline for comparison, while the 0% B. pumilus mixes contained jute fibres (treated or untreated) but no bacterial cells, thereby allowing evaluation of the effect of fibre treatment independently of bacterial activity.

#### Bacterial Preparation and dosage

Bacillus pumilus was cultured for 24 h in nutrient broth at 30 °C, harvested by centrifugation, washed, and resuspended, with cell concentration adjusted using OD₆₀₀ measurements and confirmed by plate-counting to ensure ≥ 95% viability, after which three suspensions were prepared for mixing water substitution: 1.5 × 10⁸ cells/mL (B1.5), 12 × 10⁸ cells/mL (B12), and 24 × 10⁸ cells/mL (B24)^27^.

#### Fibre–bacteria immobilization

Jute fibres were treated in 5% NaOH, rinsed, dried, and soaked in *B. pumilus* suspension for 24 h to promote bacterial adhesion and improve fibre–matrix interaction^30^.

#### Concrete mixing and curing

**Fig. 4:**
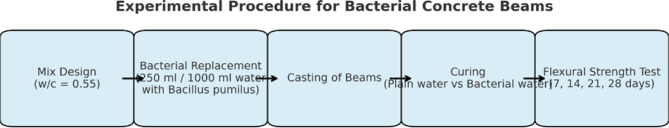
Flow chart for bacteria concrete beams.

Bacterial concrete was produced by replacing part of the mixing water with the prepared suspensions. Table 2 shows the mix proportions. Ingredients were mechanically mixed until uniform. Each mix produced twelve (12) prismatic beams (150 × 150 × 500 mm), with the following per-batch quantities: 45.54 kg cement, 97.98 kg fine aggregate, 189.6 kg coarse aggregate, and 25.08 L total water (bacterial or plain). Moulds were cleaned, oiled, filled in layers, and compacted by tamping. Beams were covered for 24 h, demoulded, and cured in nutrient-enriched water for bacterial mixes or in plain water at 25 ± 2 °C for control and fibre-only mixes. Bacterial specimens were cured in a nutrient-broth solution (5 g/L peptone, 3 g/L beef extract, 5 g/L NaCl) formulated to maintain bacterial viability without inducing CaCO₃ precipitation. The nutrient-broth solution was renewed every 7 days to maintain concentration and prevent nutrient depletion. Specimens were allocated as 3 beams at 7 days, 3 at 14 days, 3 at 21 days, and 3 at 28 days, giving a total of 12 beams per mix. Figure 4 show the flow Chart for Bacteria Concrete Beams.

**Table 2:** Mix proportion.

Pumilus/Bacterial cells/ml	Coarse Agg (g)	Fine Agg (g)	Cement (g)
Control	189,600	97,980	45,540
B1.5 Cells/ml	189,600	97,980	45,540
B12cells/ml	189,600	97,980	45,540
B24cells/ml	189,600	97,980	45,540

### Microstructural characterization

Concrete specimens, both conventional and bacterial, were examined following ASTM E1508-12 and ASTM C1723-20 standards using Scanning Electron Microscopy (SEM) to investigate microstructural features. SEM imaging at various magnifications assessed calcite deposition and fibre–matrix interactions. Specimens exposed to increasing Bacillus pumilus concentrations and 10% sulfuric acid were inspected for surface deterioration, providing qualitative and quantitative insight into internal and surface changes in the cementitious matrix. All analyses were performed at the Civil Engineering Department Materials Laboratory, Ahmadu Bello University (ABU), Zaria, Nigeria.

### Fresh concrete properties test

#### Slump test

**Fig. 5:**
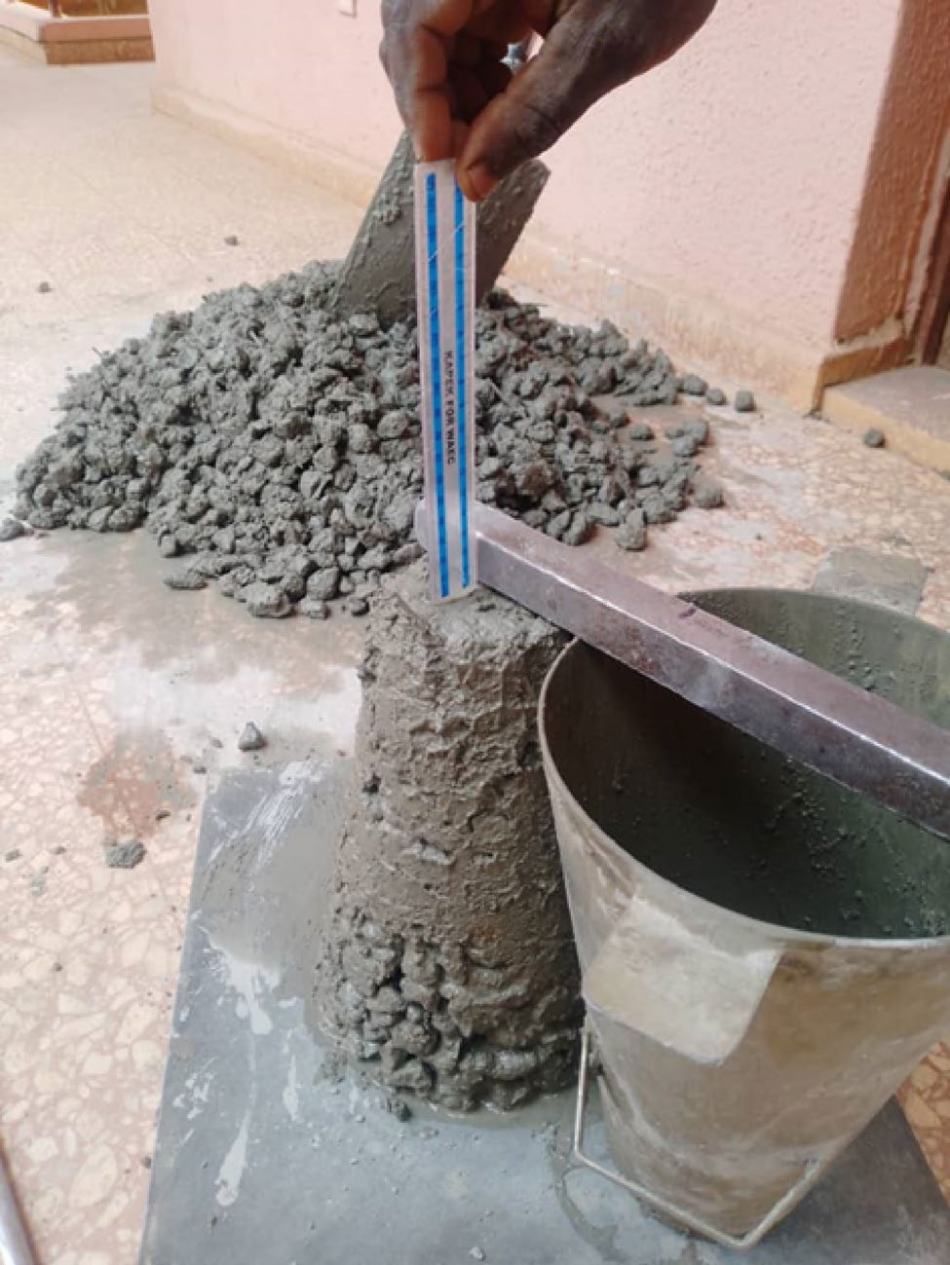
Slump test.

The workability of fresh concrete was determined using the slump test in accordance with IS 1199:1959. A standard metallic slump cone (top diameter 100 mm, base 200 mm, height 300 mm) with a 16 mm × 600 mm tamping rod and a non-absorbent base was used. Fresh concrete was filled in three layers, each compacted 25 times with the rod. After leveling the top, the cone was lifted vertically, and the slump (the vertical settlement of the concrete) was measured in millimeters. The Slump test is shown in Fig. 5.

#### Compacting factor test

**Fig. 6:**
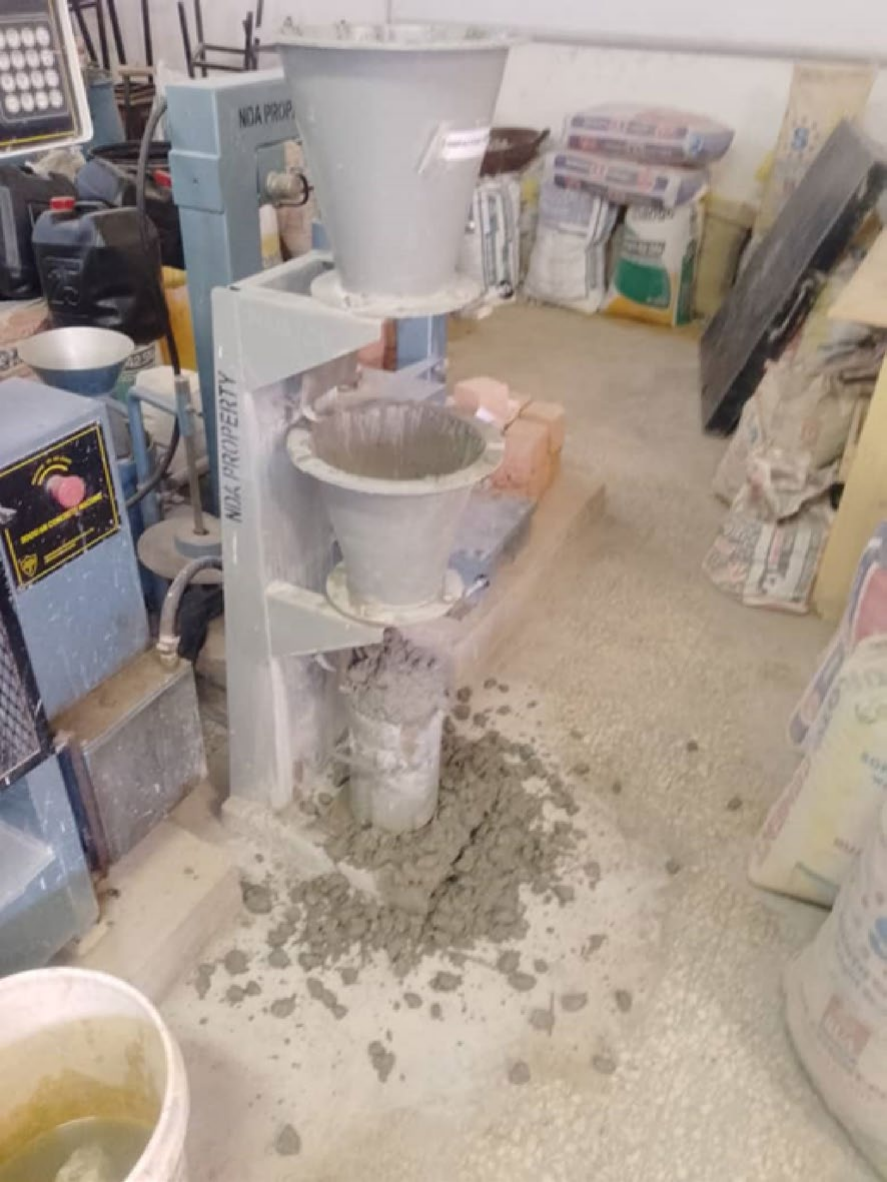
Compaction factor test.

The compacting factor of fresh concrete was determined using a compactometer in accordance with IS 1199:1959. Concrete was allowed to fall under its own weight through two conical hoppers into a cylindrical mould, and the partially compacted weight (W₁) was recorded. The mould was then fully filled in layers with mechanical compaction to obtain the fully compacted weight (W₂). Test is displayed in Fig. 6. The compacting factor was calculated in Eq. 1.

1 $$\:CF=\:\frac{{W}_{1}}{{W}_{2}}$$

### Hardened concrete test

#### Flexural strength testing

**Fig. 7:**
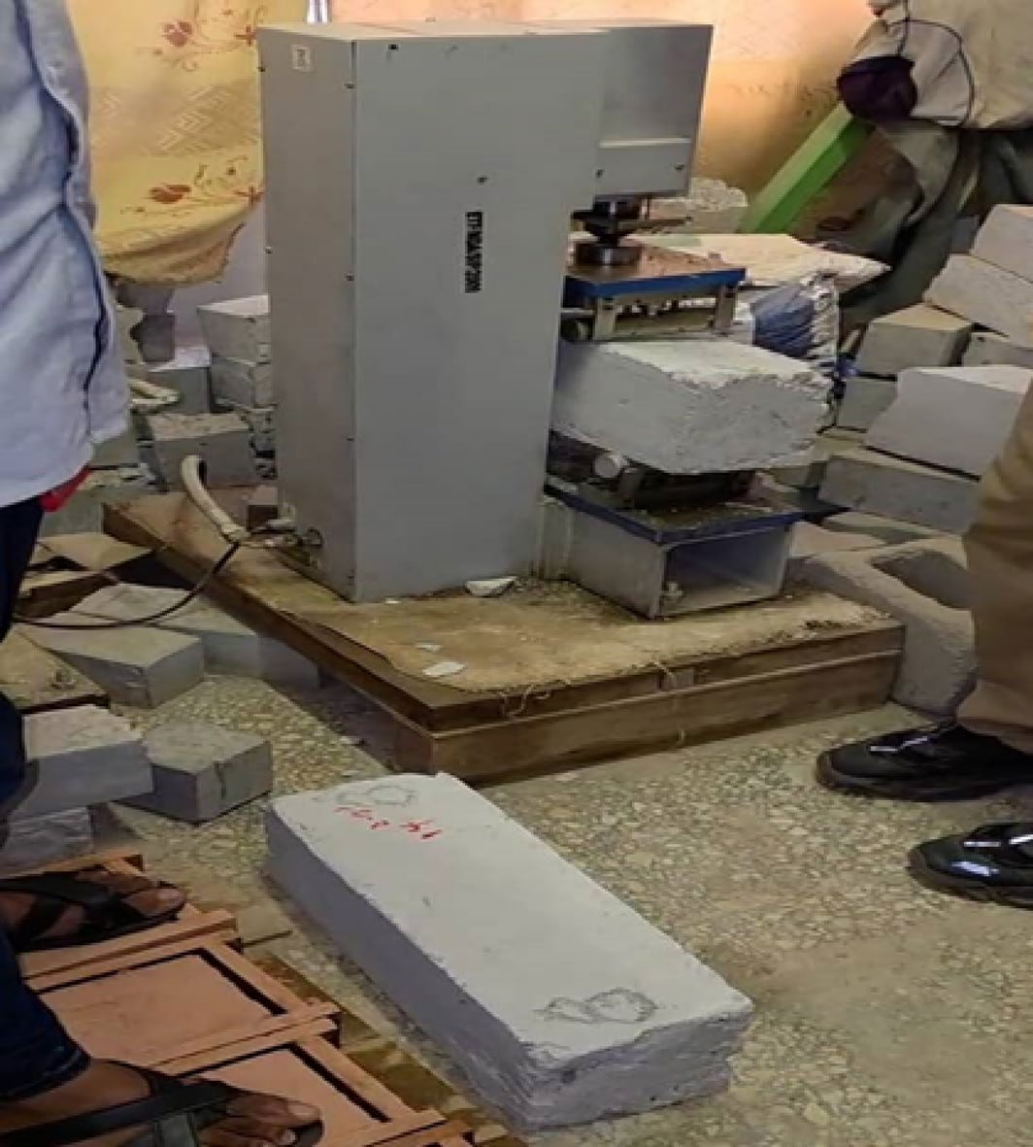
Beam specimen under loading showing mid-span deflection.

The flexural strength of conventional and bacterial concrete beams was determined using a Universal Testing Machine (UTM, 100 kN capacity) under three-point bending in accordance with ASTM C78, ASTM C293-02, and BS EN 12390-5. Prismatic beams (150 × 150 × 500 mm) were inspected for defects, and their width and depth were measured with a digital Vernier caliper (± 0.01 mm), averaging two measurements per specimen. The effective span (L) was set at 300 mm between roller supports. Specimens were positioned on the supports with their longitudinal axis perpendicular to the centrally aligned loading nose. A small preload (50 N) ensured proper contact, and the crosshead was driven downward at a controlled displacement rate of 0.45 mm/min. Load and mid-span deflection were continuously recorded until failure. Fracture modes were documented, typically occurring in the tension zone at mid-span (as shown in Fig. 7). Flexural stress was calculated using Eq. 2:

2 $$\:{\sigma\:}_{f}=\:\frac{3PL}{2b{d}^{2}}$$

Where σ_f_ = flexural strength (MPa), P is the peak load (N), L = 300 mm, b = 150 mm, and d = 150 mm.

### Durability test

#### Bacillus pumilus solution and sulfuric acid test

Durability assessment was conducted in accordance with ASTM C267 and BS EN 12390-9. Concrete beams (150 × 150 × 500 mm) were water-cured for 28 days and subsequently exposed to *Bacillus pumilus* suspensions (1.5 × 10⁸, 12 × 10⁸, and 24 × 10⁸ cells/mL) and a 10% H₂SO₄ solution. A 28-day curing period was adopted as the standard baseline for accelerated laboratory durability and bio-concrete exposure tests, as widely used in similar studies^31,32^. Each specimen was fully immersed in 1 L of medium at ambient temperature, with all biological and acid solutions renewed every 7 days to maintain concentration and prevent depletion of reactive species. Specimens were retrieved at 7, 14, 21, and 28 days. After removal from the solutions, beams were rinsed, oven-dried at 105 ± 5 °C to constant mass, and weighed to determine mass loss using Eq. 4:

4 $$\:Mass\:Loss\:\left(\%\right)=\:\frac{{M}_{0}-\:{M}_{t}}{{M}_{t}}$$

where M_0_​ is the initial mass and M_t_​ is the mass at time t. The Mass Durability Factor (MDF) was then calculated as:

5 $$\:MDF\:\left(\%\right)=100-\left(Mass\:Loss\right)\%$$

representing the percentage of mass retained relative to the initial mass.

### Statistical analysis

A statistical analysis was conducted using two-way Analysis of Variance (ANOVA). The independent variables considered were mix type (Control, B1.5, B12, and B24) and curing age (7, 14, 21, and 28 days), while the dependent variable was the flexural strength of the concrete beams. All results are reported as mean ± standard deviation based on *n* = 3 specimens per age. The two-way ANOVA approach was chosen because it allows simultaneous evaluation of the effect of multiple factors and their interaction on the response variable. Tukey’s Honest Significant Difference (HSD) post-hoc test was applied to identify which specific group pairs were significantly different. The statistical analysis was carried out using both SPSS (version 26) and Python (Statsmodels package) to ensure reproducibility and transparency. The use of Python additionally enabled automated export of results into tabular and graphical formats, which supported interpretation and visualization of group differences.

.

## Results and discussion

### Microstructural analysis

**Fig. 8:**
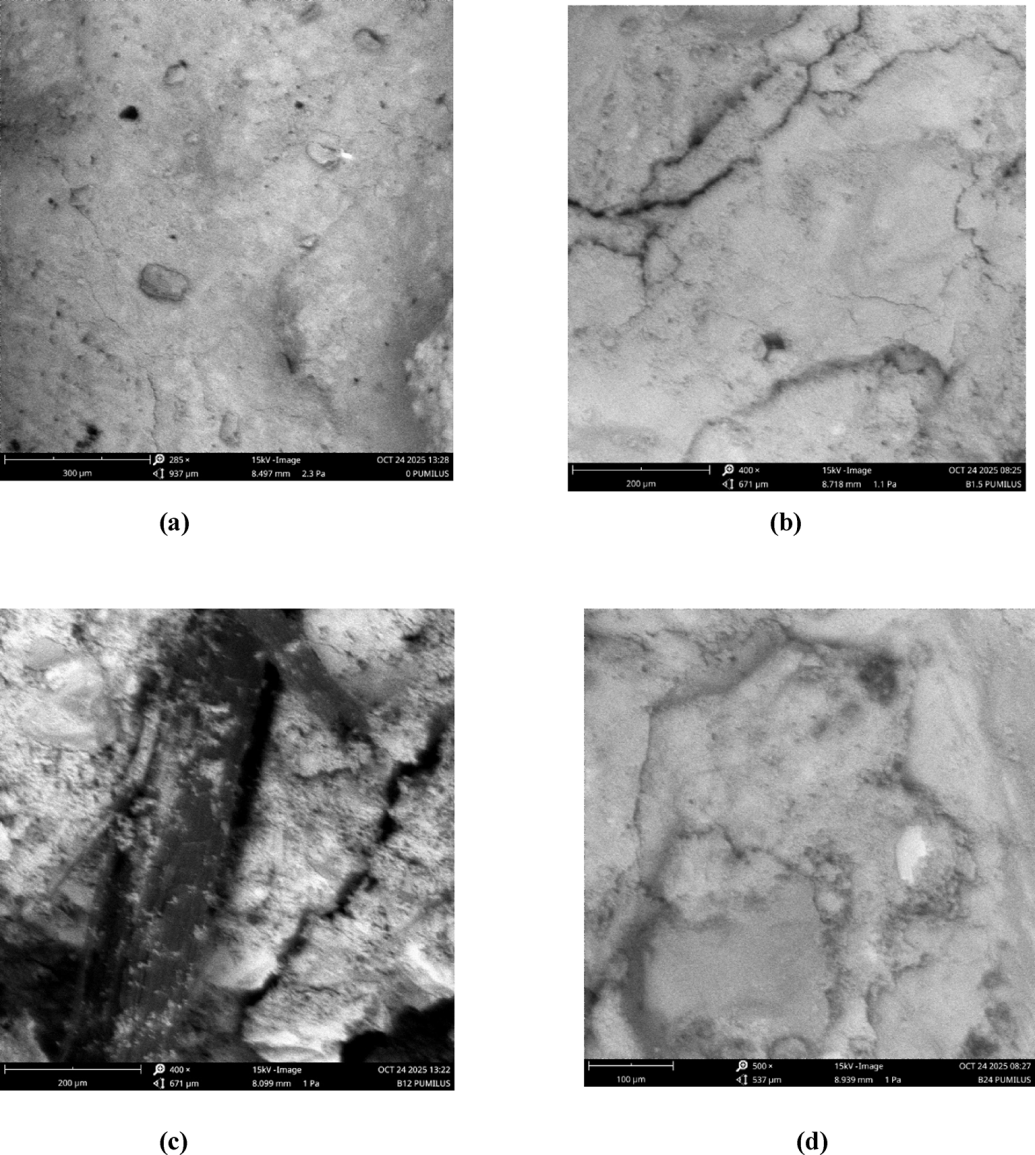
SEM micrographs of concrete specimens at 15 kV illustrating the effect of increasing *Bacillus pumilus* concentration on microstructural densification. **(a)** Control mix (0 *B. pumilus*) showing a porous matrix with unfilled microcracks. **(b)** B1.5 mix displaying scattered early calcite formations and partial crack sealing. **(c)** B12 mix exhibiting dense and uniform calcium carbonate precipitation with enhanced fibre–matrix bonding. **(d)** B24 mix showing extensive calcite agglomeration, pore filling, and the most compact microstructure, indicating maximum microbial-induced calcium carbonate precipitation (MICP).

In Fig. 8a, the control mix exhibits a porous and weakly bonded matrix with unfilled cracks and an absence of calcite precipitation, a behaviour consistent with observations by Achal et al.^33^, who noted that untreated cement matrices typically retain high porosity and poor crack-healing potential. At the B1.5 × 10⁸ level (Fig. 8b), small and sparsely distributed calcite deposits begin to emerge, causing only marginal densification; this agrees with studies indicating that low bacterial dosages initiate nucleation but are insufficient to produce structural benefits beyond minor strength gains. A more pronounced improvement occurs at B12 × 10⁸ (Fig. 8c), where dense and uniformly distributed calcite crystals effectively seal microcracks and enhance the fibre–matrix interface. This trend mirrors findings by Mekonnen et al.^34^ and Sowmya & Pannem^35^, who reported that optimal bacterial concentrations promote extensive calcium carbonate formation, resulting in significant increases in strength and durability. Finally, the B24 × 10⁸ mix shows thick, continuous calcite layers that fully bridge cracks and produce the most compact microstructure, in agreement with Nair et al.^36^, who demonstrated that high bacterial concentrations yield maximum matrix densification (as shown in Fig. 8d).

### Fresh concrete results

#### Slump result

**Fig. 9:**
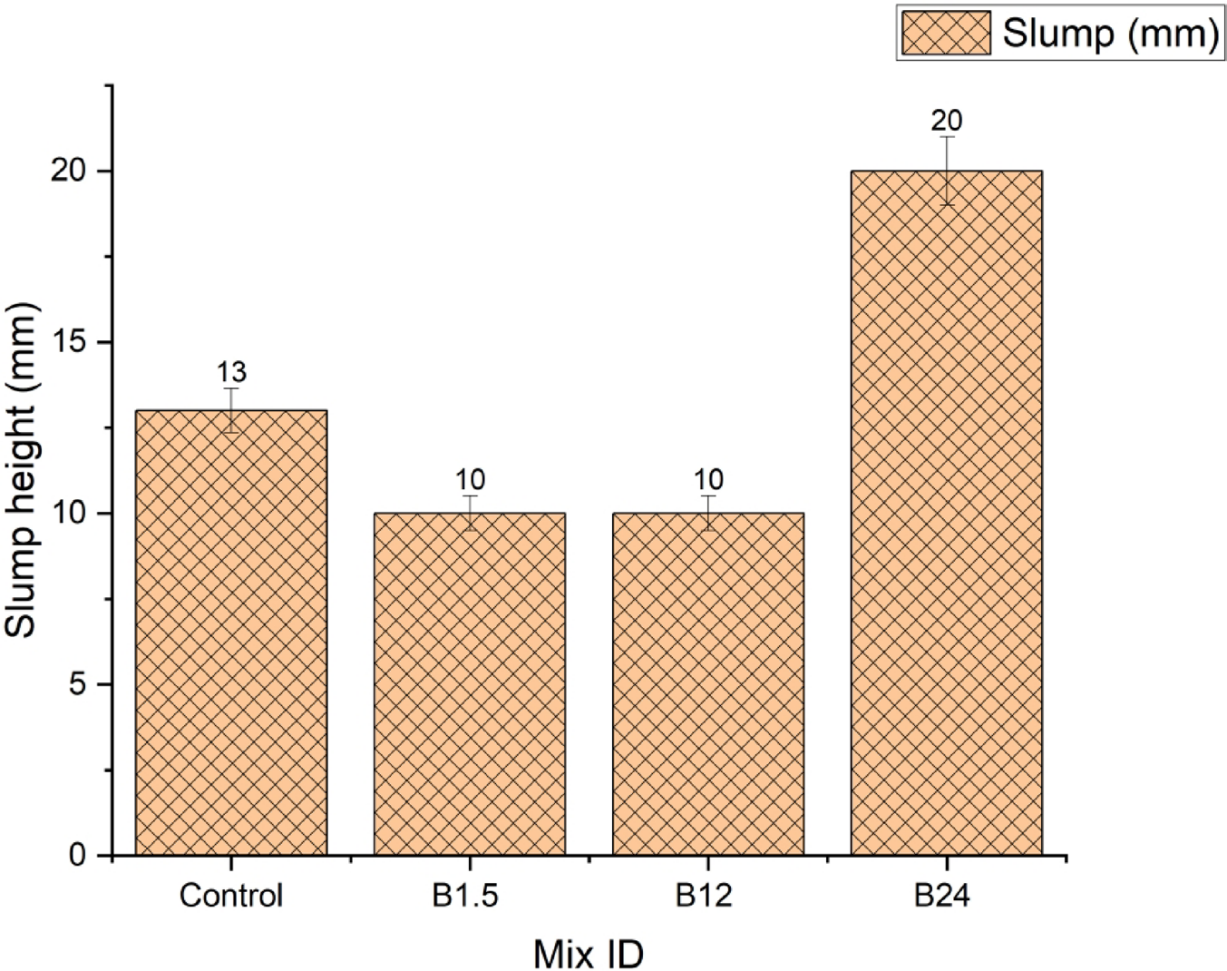
Slump result.

The slump results in Fig. 9 revealed a concentration-dependent influence of Bacillus pumilus on the workability of jute fibre reinforced concrete (JFRC). The control mix recorded a slump of 13 mm, while mixes containing B. pumilus at concentrations of 1.5 × 10⁸ and 12 × 10⁸ cells/mL both showed reduced slumps of 10 mm, indicating a slight loss in workability likely due to bacterial solids and fibre water absorption increasing internal friction. However, at the highest concentration (24 × 10⁸ cells/mL), the slump increased to 20 mm, suggesting that excessive bacterial culture or extracellular polymeric substances (EPS) acted as natural lubricants, improving flow. Similar findings have been reported by Bandlamudi et al.^37^ and De Oliveira et al.^38^, who observed that low bacterial dosages tend to stiffen the mix, while higher concentrations enhance fluidity through biofilm formation and medium effects.

#### Compactor factor results

**Fig. 10:**
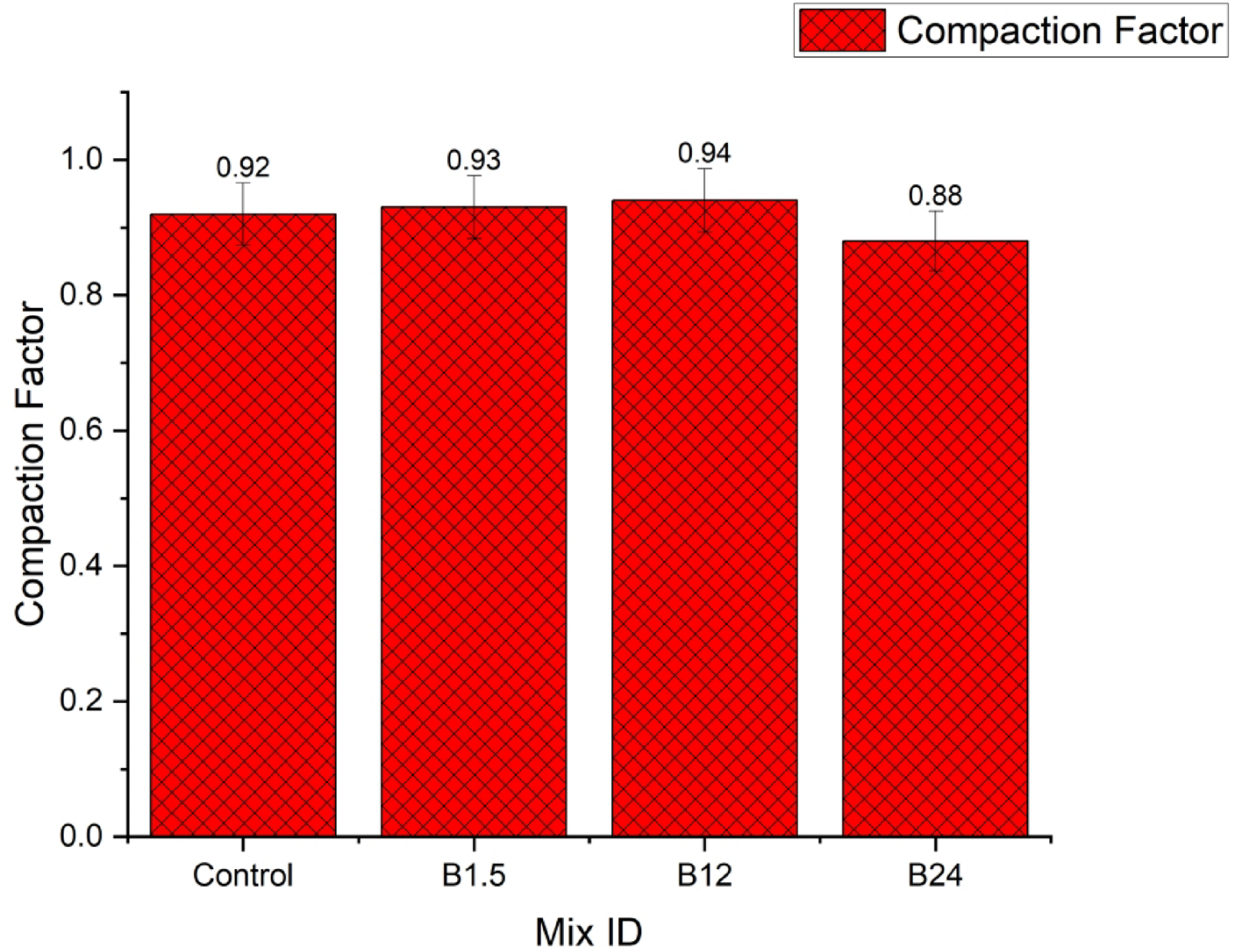
compacting factor result.

The compacting factor results in Fig. 10 revealed a concentration-dependent response to B. pumilus in JFRC: low–moderate doses slightly improved compactability (0.92 → 0.93–0.94), while the highest dose reduced compactability markedly (0.88). The reduction at B24 × 10⁸ is likely due to increased entrained air or EPS-induced viscosity and fibre clumping, which impaired particle rearrangement despite the higher slump; follow-up air-content and fresh-density tests are recommended to confirm the mechanism. The compacting factor results align with trends reported in recent studies on bacterial and fibre-reinforced concretes. Sundravel et al.^39^ observed that introducing Bacillus species at low concentrations slightly improved compactability due to enhanced particle cohesion and biofilm formation, which facilitate denser packing under compaction. Similarly, Mekonnen et al.^34^ reported that moderate bacterial dosages increase mix cohesiveness without significantly affecting flowability. However, higher bacterial concentrations often reduce compactability, as excessive extracellular polymeric substances (EPS) or air entrapment increase viscosity and hinder particle rearrangement. This behaviour is consistent with the present study, where compacting factor improved marginally from 0.92 to 0.94 at moderate bacterial levels but declined to 0.88 at the highest concentration. Furthermore, as noted by Yasin et al.^40^, jute fibres inherently reduce workability due to water absorption and internal friction, which may amplify the effects observed at higher bacterial dosages.

### Flexural strength results

#### Flexural strength of concrete with untreated jute fibre

**Fig. 11:**
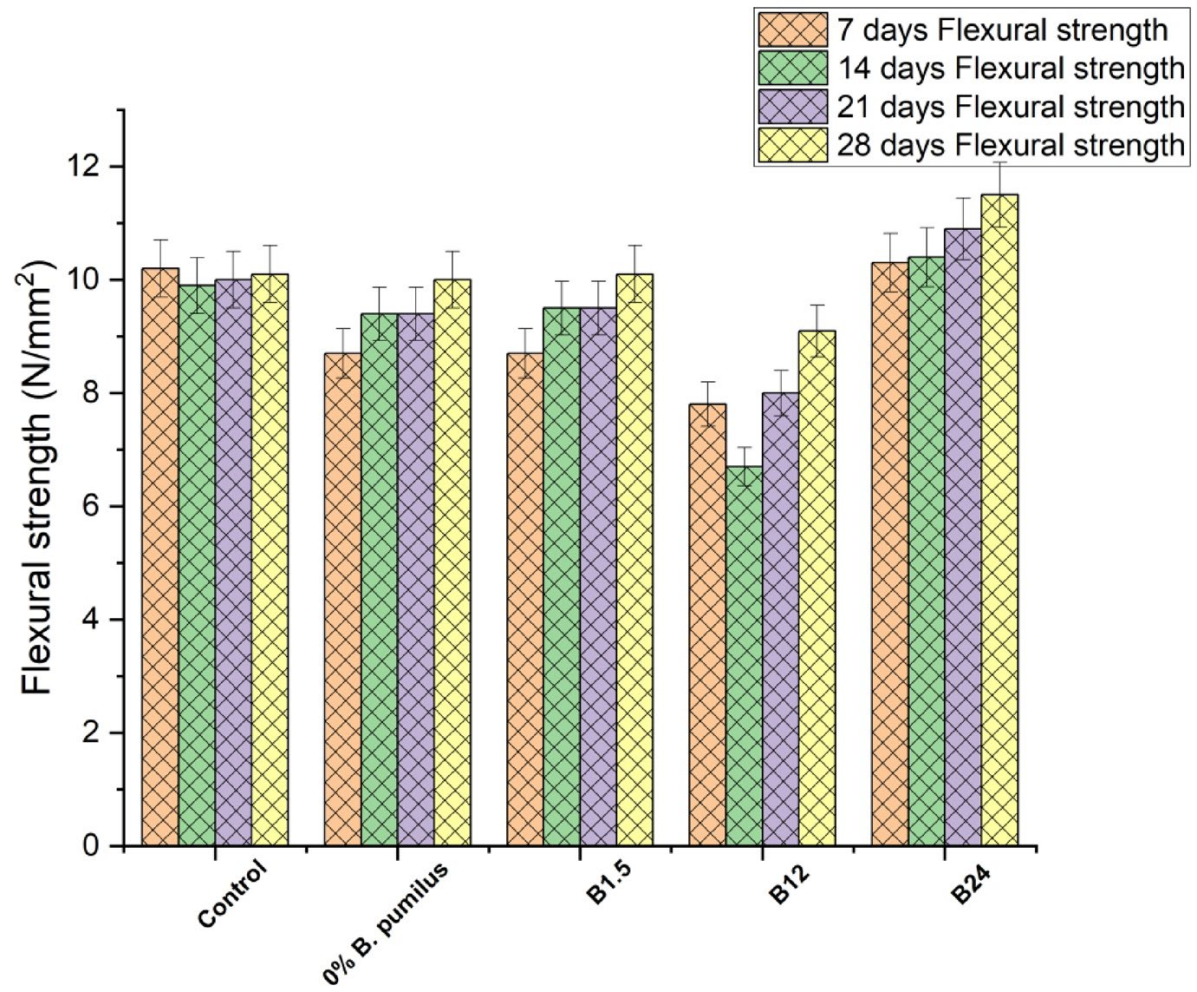
Flexural strength of concrete with untreated jute fibre.

The flexural strength results in Fig. 11 show a clear relationship between Bacillus pumilus concentration, fibre behaviour, and the microstructural features observed in the SEM images. The control mix remained around 10 MPa throughout, consistent with the SEM micrograph showing a porous, weakly bonded matrix with unsealed microcracks and no calcite deposition, which limits strength development. The untreated-jute mixes without bacteria showed lower early strength (8.7 MPa) due to fibre-induced water absorption and poor fibre–matrix bonding, also reflected in the SEM image of the control. At low and moderate bacterial dosages (B1.5 × 10⁸ and B12 × 10⁸), flexural strengths were similar or slightly lower than the control up to 21 days, aligning with SEM observations of scattered or partially formed calcite deposits that only partially bridge cracks, resulting in incomplete densification. However, at 28 days, the B24 × 10⁸ mix achieved the highest strength (11.5 MPa), about 14% higher than the control. This corresponds with the SEM image showing dense, continuous calcium carbonate deposition, superior crack sealing, and a highly compact matrix, confirming enhanced MICP-driven densification. This agrees with findings by Anjomshoa & Ramezanianpour^41^ and Nair et al.^36^, who reported improved long-term strength in bacterial concrete at optimal cell concentrations.

#### Flexural strength of concrete with treated jute fibre

**Fig. 12:**
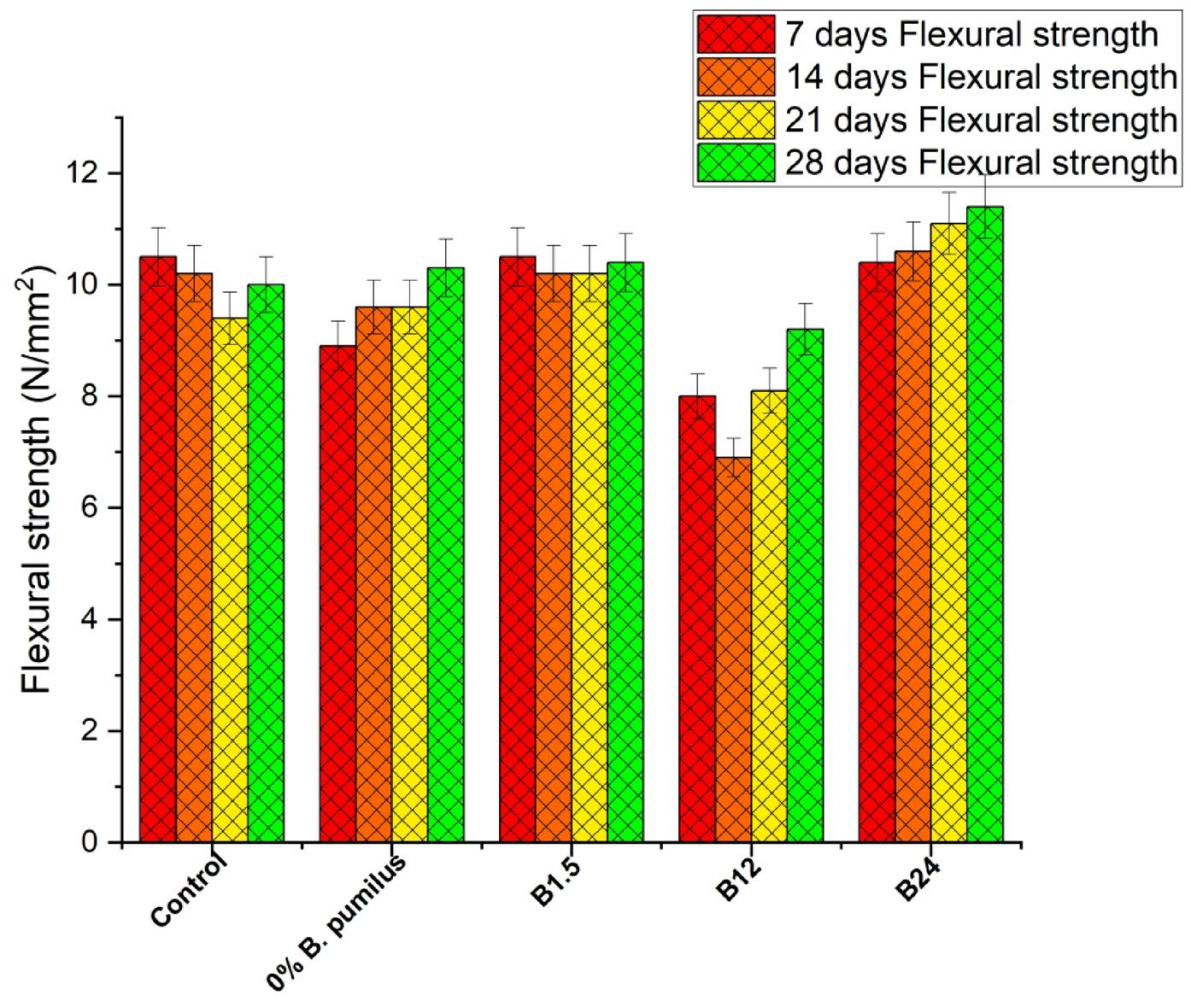
Flexural strength of concrete with treated jute fibre.

Figure 12 show that both treated jute fibre treatment and B. pumilus concentration strongly influence strength development. The treated-jute mix without bacteria performed slightly below the control at early ages (8.9–9.6 MPa) but surpassed it at 28 days (10.3 MPa), indicating that fibre treatment improved fibre–matrix bonding and reduced early-age water absorption effects. At low bacterial dosage, the B1.5 × 10⁸ mix matched the control at 7 days (10.5 MPa) and maintained stable strengths through 28 days (10.4 MPa), showing that small bacterial additions do not hinder hydration and may work synergistically with treated fibres. However, the B12 × 10⁸ mix showed reduced strength at all ages, particularly at 14 days (6.9 MPa), indicating that this intermediate concentration may have disrupted hydration or introduced microvoids before sufficient calcite precipitation occurred. The highest bacterial dosage, B24 × 10⁸, consistently produced the best performance, increasing from 10.4 MPa at 7 days to 11.4 MPa at 28 days, outperforming the control by about 14%. This suggests that treated fibres combined with high bacterial activity promote effective microbial-induced calcite precipitation (MICP), leading to improved matrix densification and enhanced fibre–matrix bonding. Overall, the result is consistent with the findings of Sowmya & Pannem^35^, who reported that the combined action of bacteria and fibres enhances both mechanical and functional properties, they found tensile strength improvements in the range of approximately 26–45%.

Table 3 presents the percentage changes in average flexural strength across curing ages (7→14, 14→21, 21→28, and overall 7→28 days) for all mixes, including the control, treated jute fibre mixes, and untreated jute fibre mixes with and without Bacillus pumilus.

**Table 3:** Percentage changes in average flexural strength across curing ages.

Mix	7→14 (%)	14→21 (%)	21→28 (%)	Overall 7→28 (%)
Control (0% pumilus, 0% jute)	6.33	1.52	6.72	15.19
0% pumilus, Treated jute	7.74	0.21	6.75	15.25
B1.5 pumilus, Treated jute	−3.24	−0.1	1.97	−1.43
B12 pumilus, Treated jute	−13.93	18.95	12.5	15.18
B24 pumilus, Treated jute	1.53	4.62	2.71	9.1
0% pumilus, Untreated jute	8.93	−0.21	6.95	16.27
B1.5 pumilus, Untreated jute	−3.49	0.2	2.2	−1.16
B12 pumilus, Untreated jute	−14.52	19.52	12.97	15.41
B24 pumilus, Untreated jute	0.2	6.65	5.68	12.93

#### Statistical analysis of flexural strength

Two-way ANOVA (Table 4) showed that both bacterial concentration and curing age significantly influenced the flexural strength of jute fibre-reinforced concrete, with a notable interaction between the factors. Tukey HSD post-hoc tests (Tables 5 and 6) revealed that mixes with higher *B. pumilus* concentrations (B12 × 10⁸ and B24 × 10⁸ cells/ml) achieved significantly higher strength than Control, fibre-only, or low-dose B1.5 mixes, whereas B1.5 showed no significant improvement. These results indicate that optimal bacterial incorporation enhances bio-calcification and matrix densification, improving mechanical performance in fibre-reinforced cementitious composites, consistent with previous studies^42,43^.

**Table 4:** Two-way ANOVA results.

Source	sum_sq	df	F	PR(> F)
C(Mix)	36.2787	8	4.07E + 28	1.51E-57
C(Age)	6.6212	3	1.98E + 28	8.50E-57
C(Mix): C(Age)	4.5151	24	1.69E + 27	7.60E-55
Residual	4.46E-28	4	nan	nan

**Table 5:** Tukey HSD post-hoc results (28 Days).

Group1	Group2	Meandiff	*p*-adj	Reject
0% pumilus, Treated jute	0% pumilus, Untreated jute	−0.13	0	TRUE
0% pumilus, Treated jute	B1.5 pumilus, Treated jute	0.07	0	TRUE
0% pumilus, Treated jute	B12 pumilus, Treated jute	−1.1	0	TRUE
0% pumilus, Treated jute	B24 pumilus, Treated jute	1.11	0	TRUE

**Table 6:** Tukey HSD post-hoc results (all curing ages).

Group1	Group2	Meandiff	*p*-adj	Reject
0% pumilus, Treated jute	B12 pumilus, Treated jute	−1.5675	0.0206	TRUE
0% pumilus, Treated jute	B12 pumilus, Untreated jute	−1.7	0.0093	TRUE
B1.5 pumilus, Treated jute	B12 pumilus, Treated jute	−2.2475	0.0003	TRUE
B12 pumilus, Treated jute	B24 pumilus, Treated jute	2.8375	0	TRUE

To clearly distinguish the individual and combined contributions of jute fibre, *Bacillus pumilus* dosage, and their interaction, the flexural results in Figs. 11 and 12 were re-analysed alongside the statistical outcomes in Tables 3, 4, 5 and 6. The influence of jute fibre alone is evident when comparing the Control mix to the 0% *B. pumilus* mixes, where fibre addition—treated or untreated—reduces early-age strength due to water absorption and delayed hydration but achieves comparable or slightly higher 28-day strength through intrinsic mechanisms such as crack bridging (Table 3). When fibre content is held constant, increasing bacterial dosage (B1.5 → B12 → B24) consistently enhances 28-day strength, confirming a dosage-dependent MICP effect driven by increased CaCO₃ deposition and matrix densification, in agreement with the microstructural patterns shown in Fig. 8c–d. The combined fibre–bacteria response exceeds the individual contributions of each mechanism, as treated fibre with B24 produces the highest 28-day strength and SEM images (Fig. 8d) show dense CaCO₃ nucleation along fibre surfaces that improves anchorage and reduces pull-out—an interaction absents in fibre-only or bacteria-only mixes. Two-way ANOVA (Table 4) confirms significant main effects for fibre condition and bacterial dosage (*p* < 0.05), while the significant Mix × Age interaction indicates that bacterial precipitation modifies fibre effectiveness rather than acting independently; Tukey HSD comparisons (Tables 5 and 6) further clarify which mix combinations differ significantly. Overall, the results show three distinct contributions to JFRC behaviour—fibre effects (early-age reduction, late-age toughening), bacterial dosage effects (MICP-driven densification), and fibre–bacteria interaction effects (CaCO₃-enhanced fibre bonding)—collectively validating the synergistic mechanism proposed.

### Durability

#### Durability results in bacillus pumilus solution

**Fig. 13:**
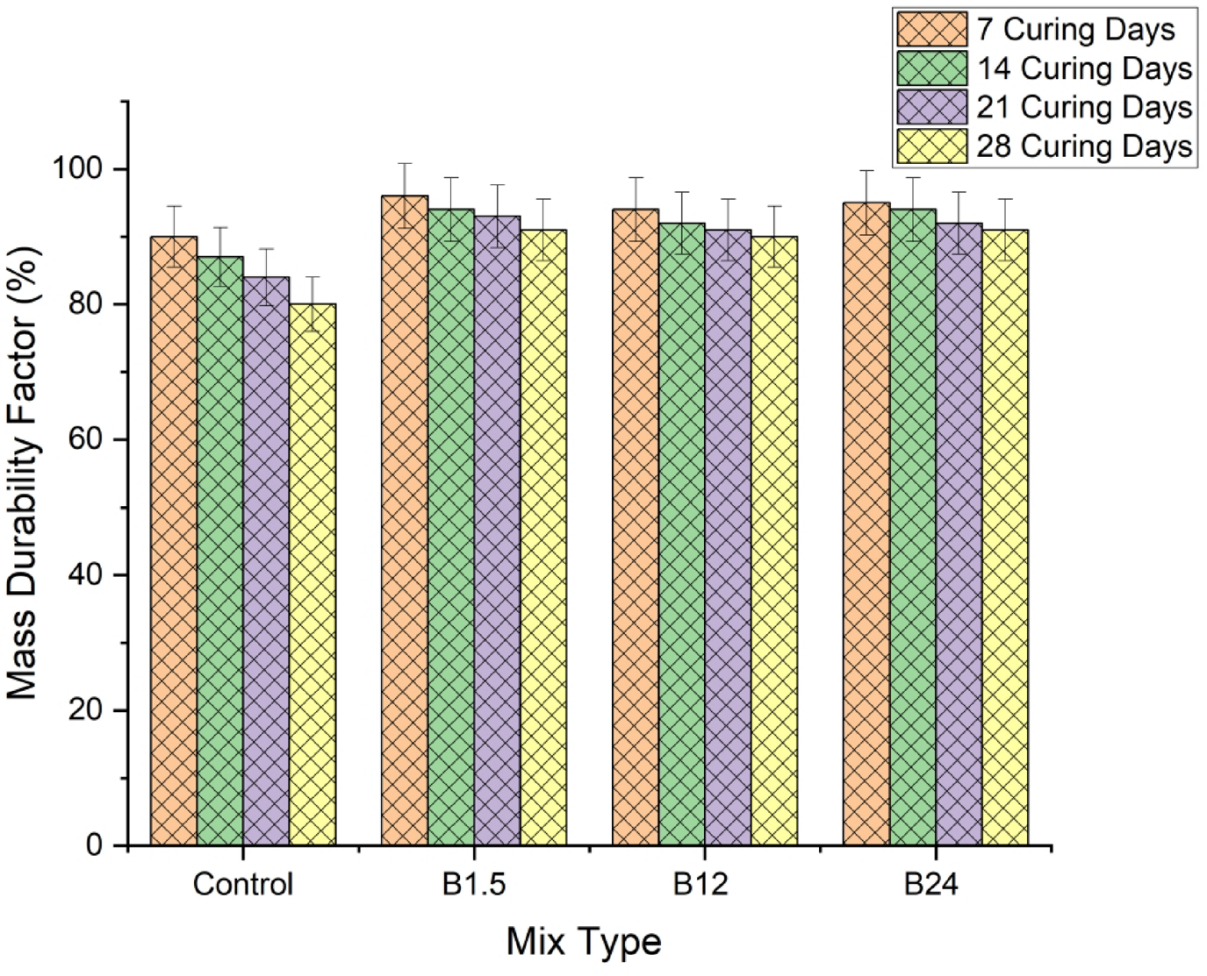
Mass durability factor result for bacillus pumilus solution.

The mass durability factor (MDF) values in Fig. 13 show a consistent decline with age across all mixes, reflecting progressive material degradation under exposure. However, bacterial incorporation significantly mitigated this loss. The control mix exhibited the steepest reduction, decreasing from 90% at 7 days to 80% at 28 days, indicating substantial mass loss and limited resistance to the aggressive environment. All bacterial mixes performed noticeably better. The B1.5 × 10⁸ mix recorded the highest MDF at every age, retaining 96–91%, which demonstrates effective early-stage microbial calcite precipitation that slows deterioration. The B12 × 10⁸ and B24 × 10⁸ mixes followed closely, each maintaining above 90% even at 28 days. Their MDF curves show gradual reductions, but the values remain significantly higher than the control, confirming that bacterial activity enhances matrix cohesion and reduces leaching. Overall, the results indicate that bacterial treatment markedly improves durability, with the B1.5 × 10⁸ mix showing the best mass retention, followed by B24 × 10⁸ and B12 × 10⁸. This trend suggests that even moderate bacterial concentrations can impart substantial resistance to mass loss, and that MICP contributes to stabilizing the cementitious matrix against chemical attack.

Table 7 shows weight loss (%) and dimensional loss (cm) of Control and B1.5 samples over 28 days. Both weight and dimensional losses increase with age, indicating progressive moisture loss and shrinkage. The Control consistently exhibits higher losses than B1.5, with 28-day values of 3.6% vs. 2.0% (weight) and 1.3 cm vs. 0.8 cm (dimensional), showing that the addition of 1.5 B. pumilus effectively reduces moisture loss and shrinkage, enhancing structural stability over time.

**Table 7:** Weight and dimensional losses of control and B1.5 samples over 28 days.

Age (Days)	Weight Loss (%) Control	Weight Loss (%) B1.5	Dim Loss (cm) Control	Dim Loss (cm) B1.5
7	1.3	0.7	0.5	0.2
14	2.1	1.1	0.8	0.4
21	3	1.6	1.1	0.6
28	3.6	2	1.3	0.8

#### Durability results in 10% H₂SO₄ solution

**Fig. 14:**
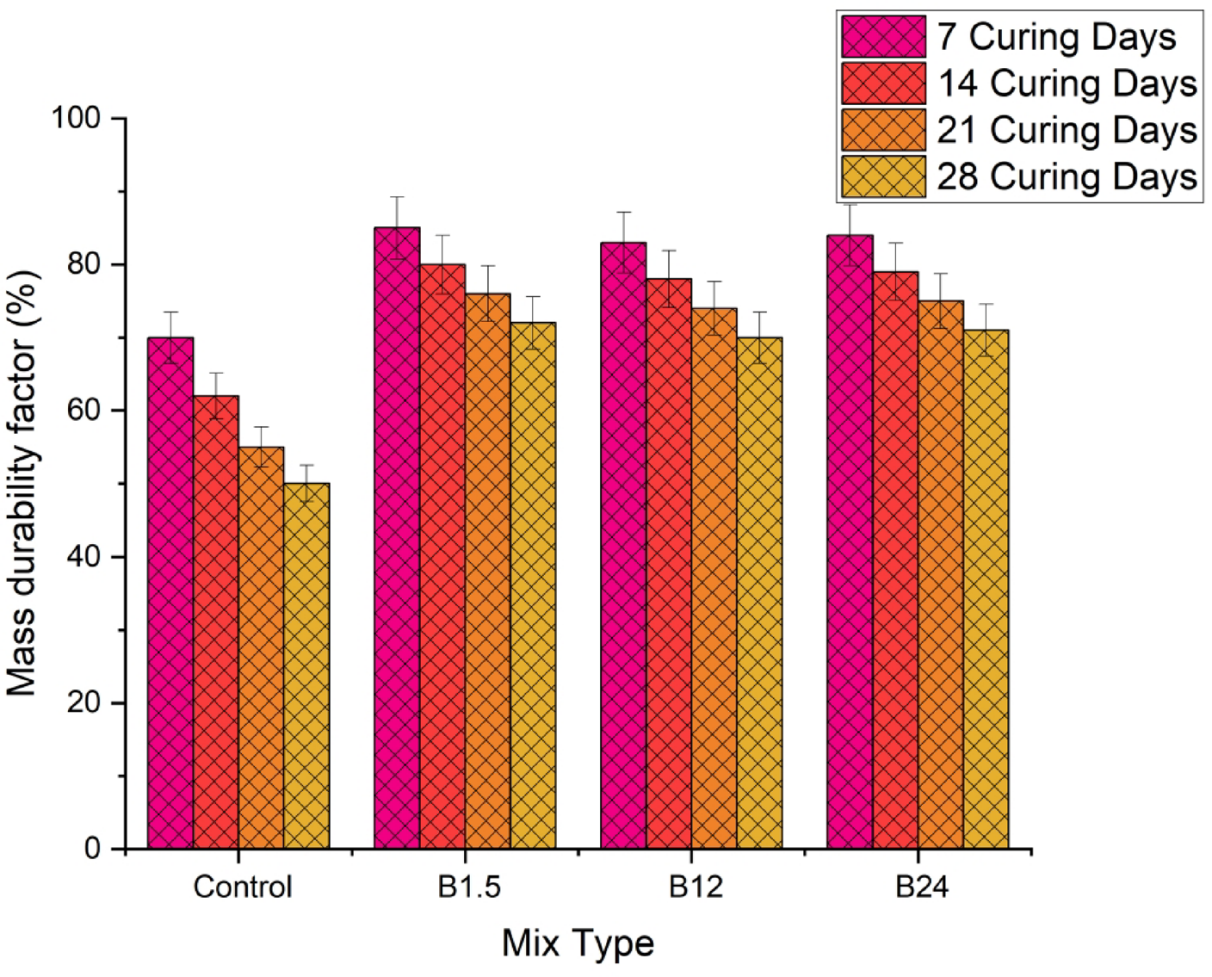
Mass durability factor result for 10% H₂SO₄ solution.

The MDF values in Fig. 14 reflect the material’s resistance to degradation in 10% H₂SO₄ solution over 28 days. The Control samples decline steadily from 70 at 7 days to 50 at 28 days, indicating significant deterioration due to acid attack. In contrast, B1.5, B12, and B24 maintain higher MDF values throughout, with B1.5 showing the highest (85 → 72), followed by B24 (84 → 71) and B12 (83 → 70). This demonstrates that incorporating B. pumilus enhances acid resistance and preserves structural integrity, while the smaller differences between B12 and B24 suggest limited additional benefit from higher bacterial content. Overall, the additives effectively mitigate acid-induced deterioration compared to the Control.

Table 8 shows the weight loss (%) and dimensional loss (cm) of Control and B1.5 samples in 10% H₂SO₄ solution over 28 days. Both weight and dimensional losses increase with exposure time, reflecting progressive acid-induced degradation and shrinkage. The Control exhibits consistently higher losses than B1.5—for example, at 28 days, weight loss is 13.2% vs. 8.1%, and dimensional loss is 2.5 cm vs. 1.6 cm—indicating that incorporating 1.5 B. pumilus significantly improves acid resistance, reducing both material erosion and structural deformation.

**Table 8:** Weight loss (%) and dimensional loss (cm) of control and B1.5 samples in 10% H₂SO₄ solution over 28 days.

Age (Days)	Weight Loss (%) Control	Weight Loss (%) B1.5	Dim Loss (cm) Control	Dim Loss (cm) B1.5
7	4.5	2.5	0.9	0.5
14	7.9	4.5	1.5	0.9
21	10.7	6.2	2	1.3
28	13.2	8.1	2.5	1.6

Although the B1.5 mix did not exhibit significant improvement in flexural strength, it consistently achieved higher MDF values than the control. This behaviour is not contradictory because flexural performance and durability are governed by different microstructural mechanisms. At low bacterial dosage, CaCO₃ precipitation is limited to early nucleation, which does not substantially strengthen the fibre–matrix interface or bridge internal microcracks; therefore, flexural strength gains remain minimal. However, even small quantities of CaCO₃ are sufficient to seal surface pores and reduce leaching, which improves mass retention and resistance to chemical attack. Consequently, the B1.5 mix demonstrates improved durability despite modest mechanical enhancement, reflecting the distinct mechanisms controlling strength development versus degradation resistance.

## Conclusion

This study examined the influence of Bacillus pumilus–induced calcium carbonate precipitation on the flexural behaviour, durability, and microstructure of jute fibre–reinforced concrete (JFRC). All research objectives were achieved through experimental testing and statistical evaluation, with the major outcomes summarised as follows:

i. Jute fibre exhibited moderate tensile strength and high cellulose content, confirming its suitability for cementitious reinforcement. The introduction of B. pumilus promoted biochemical CaCO₃ precipitation, evidenced by increased matrix density and improved fibre–matrix cohesion.
ii. B. pumilus significantly enhanced JFRC flexural strength. The mix containing 24 × 10⁸ cells/ml achieved 11.39 MPa at 28 days, an increase of 13.8% relative to the control (10.01 MPa). This improvement is attributed to microbial mineralisation, which reduced internal voids and strengthened the fibre–cement interface.
iii. Concentrations of 12 × 10⁸–24 × 10⁸ cells/ml yielded optimal performance, balancing calcite formation with workable consistency. Lower concentrations resulted in inadequate precipitation, while higher levels reduced slump by up to 35%.
iv. Bacteria-treated JFRC demonstrated superior acid resistance, retaining 20–25% more strength than the control. The improved performance is attributed to calcite filling of microcracks, reducing acid ingress and mass loss.
v. SEM results confirmed progressive microstructural improvement with increasing B. pumilus concentration, characterised by reduced porosity, denser matrix formation, and enhanced fibre–matrix bonding. The B12 and B24 mixes exhibited the most refined morphology, correlating with their superior mechanical and durability performance and demonstrating the efficacy of controlled bacterial calcite precipitation.

### Recommendations

1. High bacterial dosage (B24) is recommended for structural applications where maximum flexural strength is required, but admixtures such as plasticizers should be used to counteract reduced workability.
2. Moderate dosage (B12) may be more practical where cost or fresh concrete handling is a concern, as it offers balanced strength gains with better compaction, provided extended curing is allowed.
3. Alkali treatment of jute fibres should remain standard practice to ensure long-term durability, even though microbial precipitation can partially compensate for untreated fibre weaknesses.
4. Curing protocols should include nutrient-rich environments to sustain bacterial activity and should be extended beyond 28 days where possible to maximize precipitation and durability benefits.
5. Future studies should include long-term and field-scale durability testing under chloride attack, freeze–thaw cycles, and carbonation, as well as quantification of possible by-products such as ammonia.
6. Mechanistic studies should expand SEM/XRD quantification to correlate precipitate morphology and fibre–matrix interface strength with bulk mechanical properties.
7. Large-scale trials and cost–benefit analyses should be performed to establish the commercial viability of Bacillus-based MICP in natural fibre concrete applications.

### Limitations

This study did not include post-durability mechanical testing; therefore, strength retention after acid exposure and microbial interaction could not be directly quantified. Microbial viability and urease activity within the concrete matrix were not measured, meaning that the effectiveness of *Bacillus pumilus* was inferred from mechanical and microstructural trends rather than verified experimentally. In addition, the 10% H₂SO₄ solution used for durability assessment represents an extremely aggressive environment that does not fully reflect typical field exposure. Future studies should incorporate viability monitoring, more realistic environmental conditions, and combined mechanical–durability evaluations to improve practical relevance.

## Data Availability

The authors declare that the data supporting the findings of this study are available within this paper.
